# Estimation of absorbed dose to the kidneys in patients after treatment with ^177^Lu-octreotate: comparison between methods based on planar scintigraphy

**DOI:** 10.1186/2191-219X-2-49

**Published:** 2012-09-24

**Authors:** Maria Larsson, Peter Bernhardt, Johanna B Svensson, Bo Wängberg, Håkan Ahlman, Eva Forssell-Aronsson

**Affiliations:** 1 Department of Radiation Physics, Institute of Clinical Sciences, Sahlgrenska Cancer Center, Sahlgrenska Academy, University of Gothenburg, Sahlgrenska University Hospital, Gothenburg, SE-413 45, Sweden; 2 Department of Oncology, Institute of Clinical Sciences, Sahlgrenska Academy, University of Gothenburg, Sahlgrenska University Hospital, Gothenburg, SE-413 45, Sweden; 3 Department of Surgery, Institute of Clinical Sciences, Sahlgrenska Cancer Center, Sahlgrenska Academy, University of Gothenburg, Sahlgrenska University Hospital, Gothenburg, SE-413 45, Sweden

**Keywords:** Kidney, Dosimetry, Neuroendocrine tumor, ^177^Lu-octreotate, Scintigraphy, Conjugate-view method

## Abstract

**Background:**

Lu-[DOTA^0^, Tyr^3^]-octreotate (^177^Lu-octreotate) is used to treat neuroendocrine tumors with high somatostatin-receptor expression. ^177^Lu-octreotate is mainly excreted via the kidneys, but to some extent, accumulates in the kidney cortex due to, e.g., tubular reabsorption. Renal toxicity is one of the main limiting factors in ^177^Lu-octreotate treatment. Further knowledge of the biodistribution and dosimetry of ^177^Lu-octreotate in individual patients is needed. The aim of this study was to estimate the absorbed dose to the kidneys and compare the results obtained with planar imaging and different dosimetric methods: (1) conjugate-view (CV) method using patient-specific kidney sizes, (2) PA method, based on posterior images only, (3) CV method with reduced number of time points (CV_reduced data_), and (4) CV method using standard kidney sizes (CV_standard size_).

**Methods:**

Totally, 33 patients each received 3.4 to 8.2 GBq of ^177^Lu-octreotate up to five times, with infusion of lysine and arginine to block the renal uptake. Whole-body planar gamma camera images were acquired on days 0, 1, 2, and 7. The ^177^Lu concentration in the kidneys was determined by the CV method, and the absorbed dose was estimated with patient-specific organ sizes. Comparison to the CV method was made using posterior images only, together with the influence of the number of time points and with standard organ sizes.

**Results:**

Large interindividual variations were found in the time-activity curve pattern and in the absorbed dose to the kidneys using the CV method: 0.33 to 2.4 Gy/GBq (mean =  0.80 Gy/GBq, SD = 0.30). In the individual patient, the mean deviation of all subsequent kidney doses compared to that of the first administration was 1% (SD = 19%) and 5% (SD = 23%) for the right and left kidneys, respectively. Excluding data for day 7 resulted in large variations in the absorbed dose.

**Conclusion:**

Large interindividual variations in kidney dose were found, demonstrating the need for patient-specific dosimetry and treatment planning.

## Background

The somatostatin analog ^177^Lu-[DOTA^0^, Tyr^3^-octreotate (^177^Lu-octreotate) has been used to treat patients with somatostatin-receptor expressing neuroendocrine tumors with promising clinical results [1-4]. About one-third of the patients showed complete, or partial, tumor remission and many patients with advanced tumor burden experienced symptomatic relief.

After infusion of ^177^Lu-octreotate, ^177^Lu is mainly excreted via the kidneys but also reabsorbed and accumulated in the kidney cortex [5-10]. The mechanisms behind this accumulation are not fully understood, but studies on rats indicate that endocytosis either via the megalin-cubulin complex, or via ligand-specific endocytosis, pinocytosis, organic anion and cation transporters families, and oligopeptide transporters, are involved [5,8,10-13].

Renal toxicity is one of the main limiting factors in ^177^Lu-octreotate treatment [4,14,15]. To avoid adverse effects of ^177^Lu-octreotate and to determine the tolerance dose to kidneys, there is a need for better knowledge of the renal biodistribution, biokinetics, and dosimetry of ^177^Lu-octreotate and of relevant radiobiological and toxicity factors [4,5,11].

Detailed dosimetric calculations are time- and labor-intensive. The most commonly used methods include anterior and posterior planar scintigraphy (static images or whole body scans) and the conjugate-view method (CV method) [16]. Accurate determination of absorbed dose by the CV method requires scintigraphic data from multiple time points over a reasonably long time interval following administration of the radiopharmaceutical, accurate calibration of the gamma camera system, appropriate corrections for background activity, attenuation, and scatter, and reliable estimation of organ masses. If possible, less time-consuming routine methods should be defined with adequate accuracy in activity quantification and absorbed dose determination.

Dosimetric estimations for patients treated with ^177^Lu-octreotate have previously been published [15,17-20]. Results from these publications demonstrate variations in absorbed dose to the kidneys between clinics and between methods used within a clinic, with in general lower values obtained from SPECT images compared to planar images. Differences were also obtained using different methods based on planar images and the CV method. Since the latter method is most widely used, it is of interest to study how the choice of parameters influences the results, and also to study if simplifications can be applied to reduce the calculation time.

The aim of this study was to determine the mean absorbed dose to the kidneys in 33 patients with neuroendocrine tumors treated with ^177^Lu-octreotate using the CV method. The results were compared between patients and between treatment cycles given to the same patient. Furthermore, results from (1) the CV method (with patient specific organ sizes) were compared with those from (2) the PA method (a simplified method, based on posterior images only), (3) the CV method with reduced number of time- points (CV_reduced data_) (one time point fewer), and (4) the CV method using standard organ sizes (CV_standard size_) (to study the importance of patient-specific organ sizes).

## Methods

### Patients

A total of 33 consecutive patients with neuroendocrine tumors were included, 20 men and 13 women, aged 40 to 80 years (mean 63 years). The lesions included endocrine pancreatic tumors, gastrointestinal carcinoid tumors, and one lung carcinoid tumor (Table 1). Patients recruited had adequate bone marrow, kidney (GFR > 40 mL/min), and liver functions, and high somatostatin receptor expression by the tumor tissue, demonstrated by high tumor uptake of ^111^In-octreotide (OctreoScan^TM^, Covidien, St. Louis, USA) vs. normal tissues such as kidney and liver based on a diagnostic scintigraphic examination. The estimated ^177^Lu concentration in tumor tissue always exceeded that in the liver. Some of the patients had previously undergone hepatic artery embolization, and 11 patients were treated by chemotherapy, which was terminated at least 3 months before ^177^Lu-octreotate treatment. One patient (no. 5) had only one kidney. License for the treatment with ^177^Lu-octreotate was received for each patient individually by the Swedish National Medical Products Agency.

**Table 1:** Patient data, including age, sex, type of tumor, location of metastases, and earlier treatments

**Patient number**	**Age (years) at first treatment cycle**	**Sex**	**Tumor type**	**Metastases**	**Earlier treatments**
1	61	M	Rectal carcinoid	Abd, local, Sk	Y, Mayo
2	56	M	MC	Abd	N
3	39	F	EPT	Li	Y
4	59	F	EPT	Li, Sk	Y
5, one kidney	36	M	ICT	Retroper	Y
6	65	M	LC	Pl, Br	Y
7	53	M	EPT	Li, Pa	Y
8, Li transpl	67	M	Gluc	Li, Sk	Y, Mayo
9, Li transpl	57	F	Ins	Abd	N
10, Li transpl	46	F	MC	Sk (arm)	N
11	54	M	EPT	Abd	Y, Mayo
12	49	F	MC	Abd, Sk	N
13	54	F	Duodenal carcinoma/gastrinoma	Abd, Li	N
14	72	M	MC	Li, Abd, Th	N
15, Li transpl	46	F	Gastrinoma	Li, Abd	Y, Mayo
16	55	M	EPT	Li	Y
17	66	M	MC	Li Sk	N
18	69	M	Carcinoid	Li, Abd, Th	N
19	68	F	Carcinoid	Li	N
20	41	M	Carcinoid/tailgut cyst	Li, Sk	N
21	77	F	MC	Abd, Th	N
22	61	M	MC	Li, Abd, Th	N
23	64	F	Rectal carcinoid	Li	Y (ovarian carcinoma > 20 years ago)
24	65	M	EPT	Li, Pa	N
25	57	M	MC	Abd, Li, Supraclav	N
26	63	M	Rectal carcinoid	Li, Sk	N
27	71	M	Rectal carcinoid	Abd, Li, Sk	N
28	78	F	Carcinoid, unknown primary	Li, Abd, Pelvis	N
29	56	M	MC	Li, Abd	N
30	65	M	MC	Retroper, Orb, Med	N
31	78	F	MC	Abd, Li	N
32	75	M	MC	Li, Sk	N
33	79	F	MC	Li, Sk	N

MC, midgut carcinoid; EPT, endocrine pancreatic tumor; ICT, islet cell tumor; LC, lung carcinoid; Gluc, glucagonoma; Abd, abdomen; Sk, skeleton; Li, liver; Pl, pleura; Br, brain; Pa, pancreas; Th, thorax; Med, mediastinum; Retroper, retroperitoneum; Supraclav, supraclavicular lymph nodes; Orb, orbita; Ins, insulinoma; Mayo, chemotherapy according to the Mayo Clinic program; transpl, transplant; F, female; M, male.

### Radiopharmaceuticals and administration

^177^Lu-chloride and [DOTA^0^, Tyr^3^]-octreotate were obtained from the Nuclear Research & Consultancy Group (Petten, the Netherlands, distributed by IDB Holland, the Netherlands). [DOTA^0^, Tyr^3^]-octreotate was labeled with ^177^Lu according to the instructions by the manufacturer. The fraction of peptide-bound ^177^Lu was more than 99%, as determined by instant thin-layer chromatography with 0.1-M sodium citrate as the mobile phase.

Each patient received 3.4 to 8.2 GBq ^177^Lu-octreotate up to five times (treatment cycles), i.v. infused during 30 min (200 mL/h). The number of patients that received 1, 2, 3, 4, and 5 treatment cycles were 3, 7, 10, 12, and 1, respectively, and the average activity given at cycle numbers 1 to 5 were 7.8, 7.2, 7.2, 7.2, and 7.8 GBq, respectively. To reduce kidney uptake, the patient received an i.v. infusion with lysine 2.5% and arginine 2.5% in NaCl solution (250 mL/h) over 4 h starting 30 min before the administration of ^177^Lu-octreotate. Before lysine-arginine infusion, the patient received 3 mg granisetron (Kytril®, Roche, Genentech, Inc., South San Francisco, CA, USA) i.v. against nausea. The total number of administrations and amount of ^177^Lu-octreotate at the last administration were adjusted not to exceed a total absorbed dose to the kidneys of 28 Gy. The ^177^Lu activity administered was determined using a carefully calibrated ionization chamber (Capintec CRC®-120, Scanflex Medical AB, NJ, USA). Residual activity in the tube system was corrected for.

### Scintigraphy

Two gamma camera systems were used: Picker IRIX system (Marconi, Philips, Holland) and Millennium VG Hawkeye system (General Electric Medical Systems, Milwaukee, WI, USA), both equipped with medium energy parallel-hole collimators. Planar whole body images were usually acquired by the IRIX system, while SPECT/CT images were acquired with the Millennium system. Image processing and analysis were performed on a GENIE Xeleris workstation (General Electric Medical Systems, Milwaukee, WI, USA).

One hour, and 1, 2, and 7 days after onset of the ^177^Lu-octreotate infusion anterior and posterior whole body scintigraphy was performed (scanning speed of 10 cm/min, 20% energy-window centered over the 208-keV photon peak, 256 × 1,024 matrix, pixel size of 2.06 mm (IRIX) and 2.21 mm (Millennium)). SPECT/CT was performed at day one (step-and-shoot acquisition mode: 128 × 128 matrix, 4.42-mm pixel size, 4.42-mm slice thickness, 180° gantry rotation, 3°/step, 30-s frame time duration, energy window setting as above). CT images were acquired using 140-kV tube voltage, 2.5 mAs, 2.6 rpm rotation speed, 256 × 256 matrix, 2.21-mm pixel size, and a 4.42-mm slice thickness. CT images were converted to 128 × 128 matrix and fused with SPECT images. The CT images were only used for the determination of the body thickness and thickness and volume of the kidney, and the SPECT images only for the localization of regions with specific or non-specific ^177^Lu uptake, overlying or present close to the kidneys.

### Calibration of the gamma cameras

The sensitivity, *k*, and the effective linear attenuation coefficient, *μ*, for ^177^Lu were determined for the two gamma cameras [16]. A ^177^Lu source (25 ml, 330 MBq) was positioned at 0.5 to 19-cm depth in a polystyrene phantom 1.05 g/cm^3^. Static images were acquired for 300 s, using the same matrix, pixel size and energy settings as for the whole body images. The number of counts in a region-of-interest (ROI) around the source in the images was determined and plotted versus the depth. An exponential curve was fitted to the data points; *k* was determined as the extrapolated intersection with *y*-axis and *μ* was obtained from the exponential constant of the fitted curve [16,21]. For the IRIX and Millennium systems, *k* was 10.9 cps/MBq and 7.66 cps/MBq, and *μ* was 0.119 and 0.118 cm^−1^, respectively.

### Dosimetry

For each treatment cycle, ROIs were drawn around each kidney on the posterior images from the time point when the kidneys were best visualized (day 1 or 2). These ROIs were then used for all posterior images acquired at the other time points and then mirrored and used for anterior images. In all but one patient, high ^177^Lu activity in the liver, spleen, intestines, and/or tumor tissue was overlapping one or both kidneys based on SPECT images and visual analysis of planar images. Then, small ROIs were also drawn over part of the kidney without overlap, and the number of counts was extrapolated to include the entire kidney, assuming similar counts per pixel.

For background subtraction, a small circular ROI was placed adjacent to each kidney. The mean counts/pixel was determined and scaled to a body thickness excluding the kidney thickness (using the CT image, and assuming homogeneous background activity concentration within the volume of interest defined by the background ROI) and then subtracted from the mean counts/pixel in the kidney ROI [21,22]. The ^177^Lu activity in each kidney was determined in four ways: (1) by the CV method using anterior and posterior data with patient-specific organ sizes [16], (2) using only the posterior data (PA method) with patient-specific organ sizes, (3) by the CV_reduced data_ method with patient-specific organ sizes with reduced number of time points (one less than in method (1)), and (4) by the CV_standard size_ method estimated with standard organ sizes.

For each time point, the activity in the kidney, *A*, was determined according to the CV method:

(1) $$\;A=\sqrt{\frac{R_{A}R_{P}}{e^{-\mu T}}}\frac{\mu x}{k\left(e^{\mathrm{\mu x}/2}-e^{-\mu x/2}\right)}\text{,}$$

where *R*_A_ and *R*_P_ are the background-corrected (usually extrapolated, described above) number of counts in the kidney ROI from the anterior and posterior images, respectively. *T* is the anterior-posterior body thickness at the kidney level, and *x* is the anterior-posterior kidney thickness. *k* is the gamma-camera sensitivity, and *μ* is the mean linear attenuation coefficient for ^177^Lu.

The activity in the kidney using PA method:

(2) $$\;A=\frac{R_{\text{P}}}{e^{-\mu a}}\frac{\mu x}{k\left(1-e^{-\mu x}\right)}\text{,}$$

where *a* is the distance from the back of the patient to the posterior edge of the kidney.

A mono-exponential function was fitted to the measured, decay corrected time-activity data for each kidney, with a mean fit of *R*^2^ of 0.94 ± 0.13 (SD). Two patients (nos. 15 and 27) had a somewhat poorer fit *R*^2^ < 0.5. The cumulated activity, the number of disintegrations from the time of start of infusion (*t* = 0) to infinity, *∞*, was then calculated

(3) $$\;{\tilde{A}}_{\text{kidney}}=\int_{0}^{\infty }A_{0}e^{-\lambda_{\text{eff}}\cdot t}dt=\frac{A_{0}}{\lambda_{\text{eff}}}$$

where *A*_0_ is the activity at time *t* = 0, and *λ*_eff_ is the effective decay constant for the kidney.

The volume of each kidney was estimated from the CT images by approximation by an ellipsoid, giving the length, width, and thickness of the kidney. The mass was calculated assuming the tissue density 1.05 g/cm^3^[23].

The mean absorbed dose for each kidney was determined

(4) $$\;{\bar{D}}_{\text{kidney}}={\tilde{A}}_{\text{kidney}}nE\frac{\phi }{m_{\text{kidney}}}\text{,}$$

where *nE* is the mean energy emitted by electrons per decay (147 keV for ^177^Lu) [24-26], *ϕ* is the absorbed fraction (assumed to be 1 for the electrons) [27-29], and *m*_kidney_ is the mass of the kidney.

To study the necessity of data points-in-time, the mean absorbed dose for each kidney per administered activity (*D*/*A*_administered_) was determined when omitting one of the data points (1 h, 1 day, 2 days, or 7 days) and compared with *D*/*A*_administered_ based on data from all time points. In this sub-study, data from 9 treatment cycles (in eight patients) were not included, since the patients were not examined at all time points at those treatment cycles due to their medical condition.

For comparison between dosimetric estimations using patient-specific kidney size (mass and thickness) and standard kidney size, 10 of the patients (four female and six male patients) with different gender and kidney sizes were chosen, including patients with the smallest and largest kidneys. *D*/*A*_administered_ was determined using the CV method with standard masses of the kidneys (310 g for men and 275 g for women, both kidneys, taken from ICRP 89) and body sizes (20 cm trunk and 6 cm kidney thickness for men; 18 and 5 cm, respectively, for women, adapted from MIRD Pamphlet No. 5 [23,30]).

The contribution from activity in the remainder of the body to the absorbed dose to the kidney was estimated using the whole-body cumulated activity (assumed to be uniformly distributed and excluding the renal cumulated activity) and whole-body-to-kidney *S* values for ^177^Lu from RADAR/OLINDA [31].

### Statistical analysis

Statistical analyses and linear regression analyses were performed using SPSS PASW Statistics 18 (SPSS Inc, Chicago, IL, USA). Differences between groups were analyzed using Student's *t* test; *p* values < 0.05 were considered statistically significant.

## Results

When estimating the ^177^Lu activity in the kidneys from the planar images, regions with high ^177^Lu activity within organs and tissues overlapping the kidney ROIs (mainly the liver, spleen, intestines, and paraortic lymph node metastases) were excluded. Based on all total kidney ROIs defined, the area of ‘overlapping tissues’ varied from 0% to 90% of a ROI, with typical values of 50% to 70% (in 65% of the kidney ROIs).

### Time-activity curves

**Figure 1:**
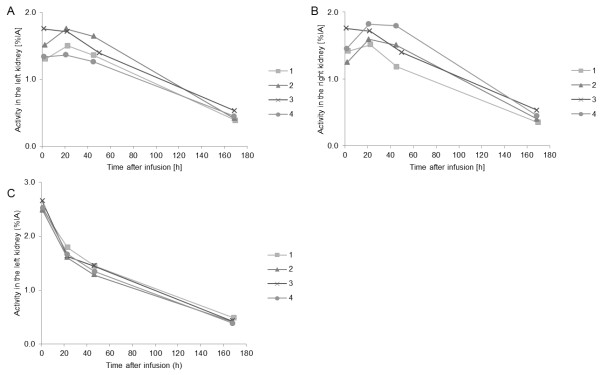
**The**^**177**^**Lu activity in the right and left kidneys given as percent of injected activity.** The ^177^Lu activity in the right and left kidneys given as percent of injected activity (%IA) for (**A**) and (**B**) patient no. 8 (left and right kidneys, treatment cycles 1 to 4) and (**C**) for patient no. 24 (left kidney, treatment cycles 1 to 4).

There were differences in the shapes of the time-activity curves of the kidneys for different patients, also among different treatment cycles in the same patient (Figure 1). There were two main patterns, either with a maximum value at the first time point studied (1 h) or at the second or third time points (day 1 or day 2). The figure also demonstrates the differences between right and left kidneys and differences among treatment cycles (Figure 1A). In two of the patients (nos. 11 and 20), the uptake phase differed between the right and left kidneys.

### Mean absorbed dose per unit administered activity

**Figure 2:**
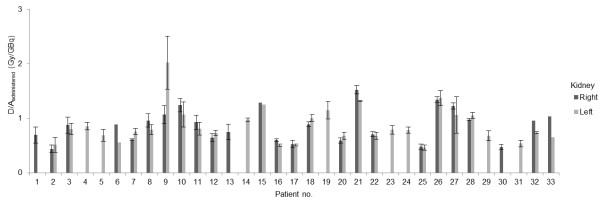
**The mean absorbed dose per unit administered activity.** The mean absorbed dose per unit administered activity, to the right and left kidneys, respectively, for all 33 patients included in the study. Results are given as mean values of all treatment cycles. Patient nos. 6, 15, and 33 only received one treatment cycle, and for patient no. 32 data for the right kidney could only be obtained from one treatment cycle. Patient no. 5 had only one kidney. For patient nos. 1, 4, 13, 14, 19, 23, 24, 29, 30, and 31 only one kidney dose could be evaluated due to high uptake in overlapping and adjacent tissues (see text). Error bars indicate ± 1 SD.

The mean absorbed dose per unit administered activity to the right and left kidneys, respectively, for all 33 patients included in the study are shown in Table 2 and Figure 2. The results show a large individual variation, with absorbed dose per unit administered activity of 0.33 to 2.4 Gy/GBq per treatment cycle for one kidney (mean value 0.80 Gy/GBq; SEM = 0.02, SD = 0.30). Considering treatment cycle 1 only, absorbed dose per unit administered activity was 0.38 to 1.7 Gy/GBq (mean value 0.84 Gy/GBq; SEM = 0.04, SD = 0.29). Patient no. 5 (with only one kidney) did not differ considerably in absorbed dose per unit administered activity to the single kidney (mean of three treatment cycles 0.69 Gy/GBq (SD = 0.11)) compared to that in patients with two kidneys.

**Table 2:** The mean absorbed dose per administered activity

**Patient number**	**First treatment cycle**	***D*/*A***_**administered**_**(Gy/GBq)**				
	**Kidney size/mass/AP thickness (cm/kg/cm)**			**Treatment cycle**		
		**1**	**2**	**3**	**4**	**5**
1	Right (5.5/0.129/27)	0.68	0.87	0.72	0.51	ND
	Left	NA	NA	NA	NA	ND
2	Right (6.8/0.168/17)	0.38	0.48	0.45	0.37	0.52
	Left (5.7/0.194/17)	0.43	0.70	0.57	0.52	0.33
3	Right (6.6/0.190/19)	1.09	0.76	0.82	0.84	ND
	Left (5.27/0.154/19)	0.91	0.68	0.76	0.86	ND
4	Right	NA	NA	NA	NA	ND
	Left (4.3/0.113/21)	0.86	0.77	0.84	0.95	ND
5	Right	NP	NP	NP	NP	NP
	Left (6.4/0.234/23)	0.74	0.57	0.76	ND	ND
6	Right (5.4/0.135/23)	0.89	ND	ND	ND	ND
	Left (5.6/0.137/22)	0.56	ND	ND	ND	ND
7	Right (6.6/0.190/24)	0.60	0.60	0.62	ND	ND
	Left (5.8/0.161/25)	0.78	0.79	0.69	ND	ND
8	Right (5.5/0.123/22)	0.60	0.60	0.62	1.1	ND
	Left (5.0/0.152/23)	0.78	0.79	0.69	0.74	ND
9	Right (5.4/0.135/16)	0.96	1.2	ND	ND	ND
	Left (3.0/0.054/18)	1.7	2.4	ND	ND	ND
10	Right (5.9/0.138/18)	1.2	1.3	ND	ND	ND
	Left (5.6/0.149/17)	0.91	1.2	ND	ND	ND
11	Right (5.4/0.157/20)	0.77	0.94	0.92	1.1	ND
	Left (6.3/0.177/20)	0.69	0.73	0.94	0.87	ND
12	Right (6.3/0.137/16)	0.73	0.61	0.59	ND	ND
	Left (5.8/0.168/17)	0.74	0.68	0.78	ND	ND
13	Right (5.1/0.131/19)	0.59	0.87	0.79	ND	ND
	Left (5.1/0.107/20)	NA	NA	NA	ND	ND
14	Right (3.7/0.087/18)	NA	NA	NA	ND	ND
	Left (4.7/0.088/19)	0.94	1.0	0.97	ND	ND
15	Right (5.7/0.159/19)	1.3	ND	ND	ND	ND
	Left (5.2/0.188/18)	1.3	ND	ND	ND	ND
16	Right (6.5/0.215/25)	0.63	0.59	0.61	0.56	ND
	Left (6.3/0.231/26)	0.52	0.52	0.47	0.51	ND
17	Right (5.6/0.187/23)	0.50	0.47	0.50	0.63	ND
	Left (6.4/0.215/24)	0.49	0.52	0.50	0.52	ND
18	Right (5.3/0.132/17)	0.85	0.88	0.94	ND	ND
	Left (5.2/0.117/19)	1.0	1.1	1.0	ND	ND
19	Right	NA	NA	NA	ND	ND
	Left (4.5/0.094/21)	1.2	0.97	1.3	ND	ND
20	Right (5.6/0.184/23)	0.66	0.56	0.57	0.57	ND
	Left (5.6/0.153/23)	0.77	0.67	0.68	0.61	ND
21	Right (5.9/0.091/16)	1.5	1.6	ND	ND	ND
	Left (4.7/0.098/18)	1.3	1.3	ND	ND	ND
22	Right (5.6/0.162/23)	0.73	0.74	0.73	0.64	ND
	Left (5.6/0.194/24)	0.74	0.72	0.66	0.60	ND
23	Right	NA	NA	NA	ND	ND
	Left (4.7/0.124/20)	0.70	0.83	0.84	ND	ND
24	Right	NA	NA	NA	NA	ND
	Left (5.6/0.167/24)	0.86	0.74	0.80	0.73	ND
25	Right (5.9/0.186/24)	0.55	0.48	0.44	0.46	ND
	Left (5.9/0.209/26)	0.52	0.47	0.40	0.46	ND
26	Right (7.0/0.189/32)	1.3	1.4	ND	ND	ND
	Left (5.5/0.145/30)	1.5	1.3	ND	ND	ND
27	Right (5.6/0.156/23)	1.2	1.3	1.2	ND	ND
	Left (6.1/0.200/22)	0.68	1.2	1.3	ND	ND
28	Right (4.4/0.111/18)	1.0	1.0	ND	ND	ND
	Left (5.1/0.158/19)	1.1	1.0	ND	ND	ND
29	Right	NA	NA	NA	NA	ND
	Left (5.6/0.181/21)	0.59	0.67	0.65	0.81	ND
30	Right (6.1/0.192/21)	0.51	0.48	0.43	ND	ND
	Left	NA	NA	NA	ND	ND
31	Right	NA	NA	ND	ND	ND
	Left (4.9/0.118/18)	0.58	0.49	ND	ND	ND
32	Right (6.2/0.155/24)	0.95	NA	ND	ND	ND
	Left (6.4/0.168/24)	0.72	0.75	ND	ND	ND
33	Right (5.3/0.116/23)	1.0	ND	ND	ND	ND
	Left (5.3/0.161/22)	0.65	ND	ND	ND	ND

NP, not present; NA, not analyzable; ND, not done. The mean absorbed dose per administered activity (Gy/GBq) to the right and left kidneys for each patient given for each treatment cycle (1 to 5 per patient). Patient no. 5 had only one kidney. For patients no. 1, 4, 13, 14, 19, 23, 24, 29, 30, 31, and 32, it was not possible to determine the absorbed dose to one of the kidneys for one or several treatment cycles (see text).

### Differences in mean absorbed dose to kidney for each treatment cycle

**Figure 3:**
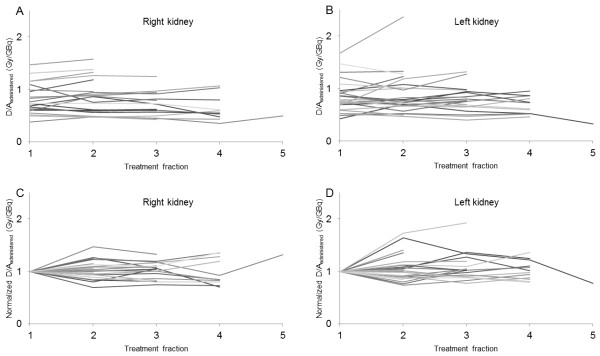
**The mean absorbed dose per unit administered activity for each treatment cycle in each patient.** The mean absorbed dose per unit administered activity given to (**A**) right kidney and (**B**) left kidney for each treatment cycle in each patient. The mean absorbed dose per unit administered activity normalized to the value for the first treatment cycle given to (**C**) right kidney and (**D**) left kidney for each patient. Each line corresponds to one patient and the different colors are used to help the reader to follow at least some of the lines to better see different patterns.

*D*/*A*_administered_ obtained for the right and left kidneys is compared in Figure 3 for each patient and treatment cycle. The mean deviation of all subsequent kidney doses from the kidney dose obtained from the first treatment cycle was 1% (range −31% to +47%) for the right kidney and 5% (range −26% to +93%) for the left kidney (Figure 3C,D). The results show no trend towards higher or lower values with increased treatment cycle number for the group, but rather a clear difference among patients.

### Comparison between the right and left kidneys

**Figure 4:**
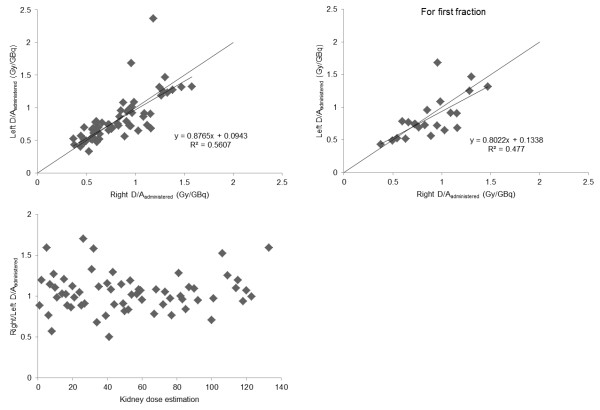
**Comparison between the mean absorbed dose per unit administered activity to the left and right kidneys.** Comparison between the mean absorbed dose per unit administered activity to the left and right kidneys for (**A**) all treatment cycles and (**B**) only the first treatment cycle. (**C**) Ratio between the absorbed dose to the right and left kidneys for all treatment cycles in the studied 22 patients.

*D*/*A*_administered_ for the right and left kidneys was compared (Figure 4). No statistically significant differences were found for the first treatment cycle (*p* = 0.43) or for all treatment cycles (*p* = 0.83) using Student's *t* test (paired, two sided).

### Comparison between CV and PA methods

**Figure 5:**
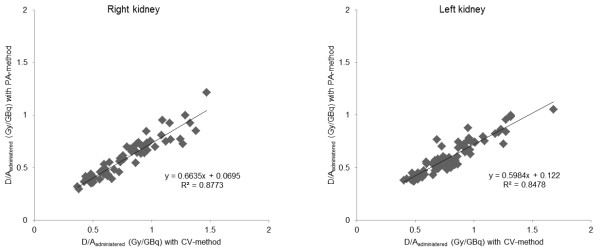
**Correlation between PA and CV methods.** Correlation between the mean absorbed dose per unit administered activity, estimated with the PA and the CV methods for the (**A**) right and (**B**) left kidneys.

*D*/*A*_administered_ was 0.60 Gy/GBq (SD = 0.17; range 0.29 to 1.2) using the PA method and 0.80 Gy/GBq (SD = 0.30; range 0.33 to 2.4) using the CV method. The correlation between the two methods was statistically significant: *p* < 0.001 for both the right and left kidneys using Student's *t* test (paired, two sided) (Figure 5).

### Importance of data points-in-time

**Figure 6:**
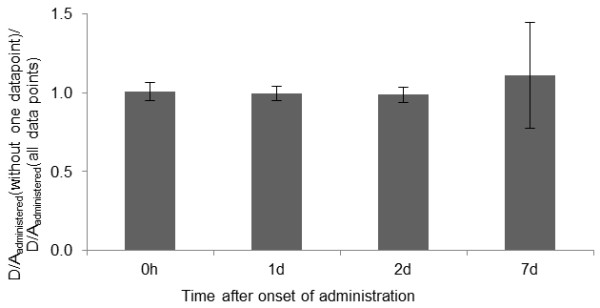
**Mean absorbed dose per unit administered activity, excluding one of the data points (CV**_**reduced data**_**method).** Mean absorbed dose per unit administered activity to the kidney determined excluding one of the data points (1 h, or 1, 2, or 7 days after administration) divided by the mean absorbed dose per unit administered activity including all data points. Error bars indicate ± 1 SD. Note that the value, when excluding the day 7, data point is underestimated since values of infinity were excluded.

The uncertainty when omitting one of the data points (1 h, 1 days, 2 days, or 7 days) was initially studied on 163 dose estimations in 32 patients, when the absorbed dose could be determined from all points-in-time. Of those 163 dose estimations, 23 were excluded, since the fitted mono-exponential curve to the time-activity curve had a positive coefficient, because day 2 had a higher uptake compared to 1 h and 1 day, resulting in infinite cumulated activity when omitting data from 7 days. The results clearly show that omitting data from 7 days gave the largest deviation in *D*/*A*_administered_ compared to *D*/*A*_administered_ from all time points, with a mean deviation by a factor of 1.1 (SD = 0.34) and a maximum deviation by a factor of 2.3 (Figure 6). It should be noted that these values when omitting results from day 7 are underestimated due to the exclusion of data with *D*/*A*_administered_ = *∞*.

### Comparison with fixed kidney mass and body size

The estimated kidney mass from CT images for the selected 10 patients was 88 to 234 g, the estimated trunk thickness was 16.7 to 26.8 cm for men and 16.4 to 21.4 for women, and the kidney thickness was 5.2 to 6.8 cm for men and 4.3 to 6.6 for women, used in the CV_standard size_ method with patient specific organ sizes (Table 2). Using the CV_standard size_ method with standard organ sizes, *D*/*A*_administered_ was underestimated by about 50% for patients with the smallest kidneys (<100 g) and overestimated by about 50% for patients with the largest kidneys (>150 g) compared to *D*/A_administered_ for the CV method with patient-specific dimensions.

### Contribution from ^177^Lu distributed in the remainder of the body

The contribution from electrons and photons from the remaining tissues (total body except the kidneys) was found to be about 2% of absorbed dose per unit administered activity to kidneys.

## Discussion

The present study focused on *D*/*A*_administered_ to kidneys in patients with neuroendocrine tumors after fractionated treatment with ^177^Lu-octreotate. The ^177^Lu activity was determined using planar whole body scintigraphy and the CV method. *D*/*A*_administered_ in our study (0.80 Gy/GBq) was similar to the results from other studies (0.65 to 1.15 Gy/GBq) (Table 3) [3,15,17-20,32].

**Table 3:** **Summary of data obtained from different studies on kidney dosimetry for **^**177**^**Lu octreotate**

	**The present study, Gothenburg**	**Valkema et al., Rotterdam[**[18]**]**	**Garkavij, et al., Lund[**[20]**]**	**Sandström et al., Uppsala[**[19]**]**	**Wehrmann et al., Bad Berka[**[17]**]**	**Cremonesi et al., Milan[**[15]**]**
Kidney protection	Lysine, arginine	Lysine, arginine	Vamin	Vamin	Lysine, arginine	ND
Number of patients	33	37	16	24	61	10
*A*_administered_/treatment cycle (GBq) mean (range)	(3.4 to 8.2)	(1.8 to 7.4)	7.4	7.4	5.4 (2.5 to 7.4)	(3.7 to 7.4)
Treatment cycles per patient	1 to 5	3 to 7	2 to 4	Only first treatment cycle	1 to 4	ND
*D*/*A*_administered_ (Gy/GBq) mean (SD)	0.80 (0.30), 0.60 (0.17)*		0.97 (0.24), 1.15 (0.29)*, 0.81 (0.21)**, 0.90 (0.21)***	0.98 (0.73), 0.71 (0.25)*, 0.65 (0.25)**	0.9 (0.3)	0.62 (median)
*D*/*A*_administered_ (Gy/GBq) range	0.33 to 2.4 Gy/GBq, 0.29 to 1.2 Gy/GBq*	7.3 to 27 Gy		2.4 to 13 Gy (right)**	0.5 to 1.7 Gy/GBq	0.45 to 18 Gy/GBq (median range)
*D*/*A*_administered_ for treatment cycle 1 (Gy/GBq) mean (range) (SD)	0.84 (0.38 to 1.7) (0.29)			0.98 (0.73), 0.71 (0.25)*, 0.65 (0.25)**		
Imaging time points	1 h, days 1, 2, and 7	ND, but several days	0.5 h, days 1, 4, and 7 (treatment cycle 1 to 2) days 1,and 4 (treatment cycle 3 to 4) SPECT at day1 and/or day 4	1 h, days 1, 4, and 7 (treatment cycle 1) SPECT at all time points	0, 3, 20, 44, and 68 h	up to 48 h
Scintigraphic method	Planar imaging	Planar imaging	Planar imaging, SPECT	Planar imaging, SPECT	Planar imaging	Planar imaging
Quantification method	CV, *PA, CT for kidney mass	CV	CV attenuation map CT for kidney mass, *different background, **SPECT to scale the time-activity curve, ***SPECT for voxel dosimetry	*SPECT organ VOI, **SPECT small VOI (4 cm^3^)	CV	CV
Dosimetry	MIRD	MIRDOSE3	MIRD	OLINDA	MIRD 16, MEDISO, OLINDA with *S* values for absorbed dose calculation	OLINDA/EXM

CV, conjugate-view method; PA, posterior-anterior view method; *A*_administered_, administered activity; D, mean absorbed dose; ND, not defined. Note that some studies give data in Gy and other in Gy/GBq, and some use mean values while other use median values. The asterisk (*) is for guiding the reader, which quantification method was used in providing the mean absorbed doses in each study compared.

When comparing the results from different studies, differences in kidney protection method, administered activity per treatment cycle, imaging technique, and methods for image analysis and dosimetry should be considered (Table 3). In one of the studies, different methods for imaging and dosimetric analysis were compared on the same patients, thus keeping the biological parameters constant: the lowest *D*/*A*_administered_ was obtained using SPECT (0.81 to 0.90 Gy/GBq) compared to planar imaging and CV method (0.97 to 1.2 Gy/GBq) [20]. Similar results were obtained in another study despite the large uncertainty associated with the planar imaging-derived dose estimates in that study [19]. Furthermore, the importance of using an optimal background correction method was demonstrated [20].

One drawback using planar images and CV method is difficulties with high and unpredictable time-dependent activity uptake in overlapping normal tissues and tumors. We tried to avoid the problem using small ROIs over parts of the kidney with no overlap, assuming homogeneous activity distribution within each kidney. We found that only one kidney could be evaluated using the whole kidney area. Due to high overlapping uptake making it impossible to outline the kidneys in the images, 34 dose estimations could not be done (eight patients for the right kidney: patient nos. 4, 14, 19, 23, 24, 29, 31, and 32 (treatment cycle 2), and three patients for the left kidney: patient nos. 1, 13, and 30). This problem would be reduced using SPECT.

*D*/*A*_administered_ varied between 0.33 and 2.4 Gy/GBq in the present study using the same method (CV method), which clearly underlines the value of individual dosimetry. We found that time-activity curve forms can differ considerably among patients and between different treatment cycles in the same patient. A similar study on 61 patients followed up to 68 h showed that the ^177^Lu activity in kidneys had a fast followed by slower clearance, with a maximal uptake of 4.0%IA [17]. This time course was seen in 21 of our 33 patients; for 12, patients we found a slower uptake phase during the first 1 to 2 days after ^177^Lu-octreotate administration. Similar differences between patients have previously been demonstrated for ^111^In-octreotide [33]. Furthermore, in two patients, we observed distinctly different time-activity curves for the right and left kidneys, maybe due to differences in kidney physiology or differences in the time-dependent activity in overlapping organs such as the intestines (see below). Ideally, multi-exponential curve fitting would have been preferable in the present study, at least for a few cases, but mono-exponential curve fitting has been routinely used in many studies and clinics, and worked well for most of the estimations in this application.

It is generally assumed that *D*/*A*_administered_ to the right and left kidneys is the same within a patient, and this assumption is used when problems with radioactivity in tissues overlying one of the kidneys occur [17]. Although we found about 12% lower values for the left kidney than for the right kidney for the patient group, this difference was not statistically significant (*p* = 0.83, paired *t* test). Monte Carlo simulations on a phantom using the CV method demonstrated that the left kidney was less influenced by radioactivity in the intestines (especially colon) than the right kidney, and that the lowest uncertainty in activity determination in an organ without overlapping activity is ±10% if appropriate attenuation and scatter corrections are used [34]. The uncertainty in kidney dosimetry for ^177^Lu-octreotate would most probably be somewhat higher, and the side difference for most patients in the present study was within ±20%, which we think reflects the total uncertainty in the present study, but values up to 70% was found (Figure 4). Due to several reasons, we believe that the absorbed dose to the kidneys should, if possible, be determined separately for each kidney. There might be individual differences in surrounding/overlapping tissues with high ^177^Lu activity concentration for the right kidney, e.g., liver with metastases, and for the left kidney, the spleen, and for both kidneys, activity in the intestines and paraortal lymph node metastases. Another reason is that the kidneys might have different functions, leading to differences in uptake and retention.

When comparing *D*/*A*_administered_ for each kidney and treatment cycle, we found no obvious change in the value with increased number of treatment cycles for the group of patients; for most of the patients, the differences were within the uncertainty (discussed above) related to ^177^Lu uptake and retention in surrounding (background) tissues. Others have found that the contribution from each treatment cycle to the total absorbed dose was uniform (difference within 10%), but for two patients, the contribution differed within 20% mainly due to differences in the kinetics [20]. In the present study, large individual variations were found for some patients (differing by up to 93% from the first treatment cycle), indicating and in some patients demonstrating a trend of increased, or decreased, kidney uptake and retention with number of treatment cycles (Figure 3). There are, however, possible sources of errors, or rather uncertainties, in the dose estimations between the treatment cycles that might overestimate the differences for a single patient: (1) The position of the ROI over the kidney might vary somewhat between subsequent imaging in a treatment cycles and/or between subsequent cycles, due to differences in patient positioning in the camera, different position and movement of the kidney (e.g., due to respiration). (2) The position of background ROIs, due to variations in radionuclide accumulation in regions surrounding the kidneys between subsequent image time points and/or treatment cycles. (3) There are uncertainties in measurement of residual activity in vials and infusion tubes and, hence, in the resulting activity administered. These results demonstrating differences in kidney dose for different cycles should be further examined in a future radiobiologically oriented study.

The results clearly demonstrate the importance of data from late time points. In cases when the maximum uptake value occurred at day 1 or 2, late time points are especially important to obtain a realistic extrapolation of the activity with time, in particular for, radionuclides with a relatively long physical half-life, such as ^177^Lu. Our finding demonstrating a large uncertainty when omitting data from day 7 (Figure 6) is also an underestimation since 23 kidney dose values were infinite and including them would give a mean value of infinity. The previous studies with no late images (only up to 48 or 68 h after infusion) reported similar *D*/*A*_administered_ levels as studies with data collected at much later points-in-time (Table 3). The reason for this is not obvious to us, since we clearly noticed the importance of data from later points-in-time for correct dose estimation in several patients.

The determination of the activity concentration in the kidneys using the CV method is rather time-consuming, and a simpler, faster method is highly desirable. Therefore, we compared the *D*/*A*_administered_ determined by the PA method with that based on the more correct CV method. We found a clear correlation between the values obtained with the two methods, but the linear regression coefficient was far from unity, demonstrating that the PA method gave too low *D*/*A*_administered_ values. The PA method requires detailed knowledge of the depth and thickness of the kidneys and assumes homogeneous activity distribution together with accurate attenuation correction. Estimations based on five patients indicate a difference of about 0.12 Gy/GBq/cm of kidney thickness (data not shown). Since the performance of the PA method was poor, CV method is preferred because the method is independent of the depth of the kidneys, and that a correct attenuation correction needed for the PA method might be complicated and require Monte Carlo simulations.

When calculating the absorbed doses to kidneys with the CV_standard size_ method using standard kidney size (MIRD5 and ICRP 89) instead of patient-specific body and kidney sizes, large differences were found for patients with small or large kidney volumes. In agreement with a study on ^86^Y/^90^Y-DOTATOC, we thus found that individual kidney volume determination is needed for a more precise estimation of absorbed dose to the kidneys [14].

## Conclusion

In conclusion, a large variation in absorbed dose per unit administered activity was found between patients (up to a factor of 8) and between administrations in the same patient (up to a factor of 2). The importance of patient-specific kidney and body sizes was demonstrated for kidney dose estimation of ^177^Lu-octreotate. There was a clear correlation between the absorbed dose determined by the simpler PA method and the more labor-demanding CV method, but the PA method gave too low values of the kidney dose. CV method is mathematically correct but assumes homogeneous activity concentration with depth within the kidney. One advantage with the CV method is that the AP position of the kidney does not influence the results. PA method is also mathematically correct and also assumes homogeneous activity concentration with depth within the kidney. The disadvantage with the method is that the AP position clearly influences the results and the AP position must be exactly defined. In most patients, the kidney axes are usually tilted compared to the back, which makes the determination of the AP position even more difficult. Therefore, the CV method is still preferred due to the higher accuracy expected. The need for late data points for better dose estimation for ^177^Lu-octreotate is also shown.

The results clearly demonstrate the importance of individualized dosimetric calculation for critical normal tissues for optimal treatment planning using ^177^Lu-octreotate. Underestimation of the actual absorbed dose to the kidneys may lead to renal failure, while overestimation of the absorbed dose would result in under-treatment of the tumor tissue and reduced treatment efficacy. To obtain accurate dosimetry, optimized activity quantification methods should be used, including methods used for scintigraphy (planar, SPECT), delineation of the organ, position of background ROI, attenuation and scatter correction, calibration, and late time points for imaging.

Further efforts should be made to define optimal but realistic dosimetric methods for the kidneys useful for routine treatment planning. The accuracy that is needed will depend on the knowledge of the tolerance dose or expected kidney toxicity at different absorbed dose (or BED) levels in the individual patient, knowledge that is still limited but needs to be obtained in future research.

## Competing interests

The authors declare that they have no competing interests.

## Authors' contributions

BW, HA, EFA, PB, and JS planned the therapeutic study, and ML, PB and EFA planned the dosimetric study. ML performed the calibrations, image analyses, and the first compilation of the data. ML and EFA made the analysis and interpretation of the data. All authors contributed to the scientific and intellectual discussion. ML drafted the manuscript. All authors read and approved the final manuscript.
